# Intraoperative Resistance to Atracurium in a Geriatric Patient on Donepezil: A Case Report

**DOI:** 10.7759/cureus.86801

**Published:** 2025-06-26

**Authors:** Niruba C, Reshmita Gandhi, Gayathri Ramesh

**Affiliations:** 1 Anesthesiology, Sree Balaji Medical College and Hospital, Chennai, IND

**Keywords:** alzheimer’s dementia, anesthesia and analgesia, anesthesia considerations, atracurium resistance, balanced anesthesia, donepezil, drug-interaction, general anesthesia, opioid-free analgesia, ultrasound-guided regional anesthesia

## Abstract

We report the case of an 84-year-old male with dementia on chronic donepezil therapy who demonstrated resistance to the non-depolarizing neuromuscular blocker atracurium during general anesthesia for orthopedic surgery. Anesthesia was successfully managed using multimodal, opioid-free techniques. The case highlights an interaction between an acetylcholinesterase inhibitor and a neuromuscular blocking agent, emphasizing the need for awareness of such interactions in elderly patients. It also underscores the importance of conducting a preoperative medication review and individualized anesthetic planning in geriatric populations.

## Introduction

Alzheimer’s disease is a common cause of dementia in elderly patients. Symptomatic treatment for dementia due to various causes is to enhance cholinergic neurotransmission in the brain by blocking the enzyme that causes the breakdown of acetylcholine. Donepezil is a cholinesterase inhibitor frequently used for this purpose [1]. However, acetylcholinesterase inhibitors should be used with caution. Although the blockade is reversible if stopped till the half-life of the drug, withdrawal before general anesthesia can lead to complications such as cognitive decline and worsening of dementia. Here, we present the case of an elderly patient with dementia taking oral donepezil who showed resistance to intravenous atracurium (a muscle relaxant used in conjunction with general anesthesia) intraoperatively.

## Case presentation

An 84-year-old man (weighing 60 kg) came to the casualty with an alleged history of a slip and fall from stairs and a sustained injury to his right arm. The patient reported no history of loss of consciousness; no ear, nose, or throat bleeding; and no head injury. The patient's son gave a history of dementia in the patient for three years and was on regular oral medications (10 mg tablet of olanzapine, 5 mg tablet of donepezil, and 10 mg tablet of quetiapine), all taken at night. He also had diabetes mellitus, for which he was on irregular medications.

The patient was diagnosed with a post-traumatic, right humerus fracture without distal neurovascular deficits. He was hemodynamically stable, with a Glasgow Coma Scale score of 12/15 (eye: 3, verbal: 4, and motor: 5). His bloodwork results were all normal. The patient and his relatives were counseled regarding surgical risks, the need for post-operative intensive care, and ventilator support. The respective consents were obtained. The patient was assessed as 3E (emergency) status according to the American Society of Anesthesiologists’ Physical Status Classification System and admitted for surgery. In the operating room, his baseline vitals were as follows: pulse rate of 80 bpm, blood pressure of 110/70 mm Hg, and oxygen saturation of 95% under room air (Table 1).

**Table 1:** Pre-operative investigations of the patient

Investigation	Value	Reference value
Blood group	A positive	
Hb	11.6 gm/dL	13-17 gm/dL
PCV	34.6	40-50
Total count	7.97×10³/µL	4×10³-11×10³/µL
Platelet	190×10³/μL	150×10³-410×10³/μL
Urea	26.09 mg/dL	17.12-49.2 mg/dL
Creatinine	0.92 mg/dL	0.9-1.3 mg/dL
Sodium	141.2 mg/dL	136-145 mg/dL
Potassium	4.01 mg/dL	3.5-5.1 mg/dL
PT	12.4 sec	13.2 sec
INR	1.08	0.9-1.1
Urine albumin	absent	
Urine sugar	absent	
HIV	negative	
HbsAg	negative	
Anti-HCV	negative	
RBS	106	140 mg/dL
Total bilirubin	0.4 mg/dL	0.3-1.1 mg/dL
Direct bilirubin	0.14 mg/dL	0.11-0.42 mg/dL
Indirect bilirubin	0.26 mg/dL	0.2-0.9 mg/dL
SGOT	14.32 IU/L	<35 IU/L
SGPT	8.33 IU/L	<45 IU/L
ALP	149.04 IU/L	56-119 IU/L
GGT	12.38 IU/L	<55 IU/L
Total protein	7.18 gm/dL	6-7.8 gm/dL
Albumin	3.83 gm/dL	3.35-5.2 gm/dL
Globulin	3.35 gm/dL	2.5-3.5 gm/dL
A/G ratio	1.14	1.2:1-2:1

**Figure 1:**
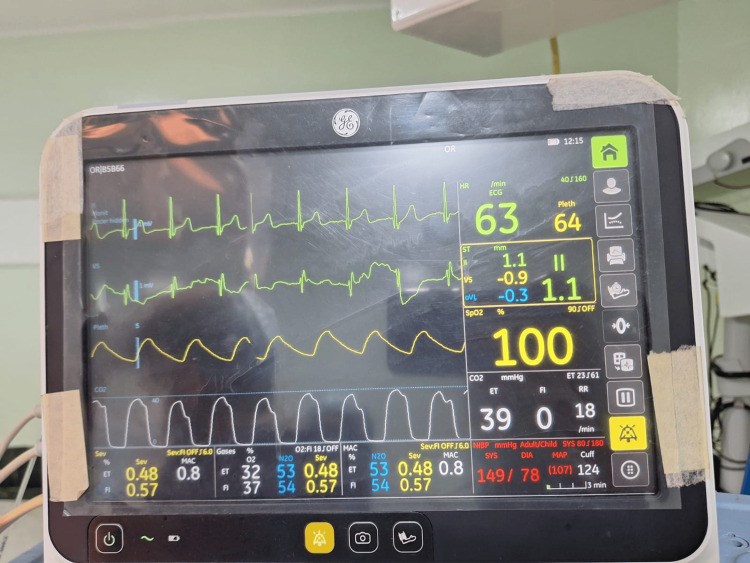
Presence of curare clefts on capnography 30 minutes after the induction dose of intravenous atracurium

**Figure 2:**
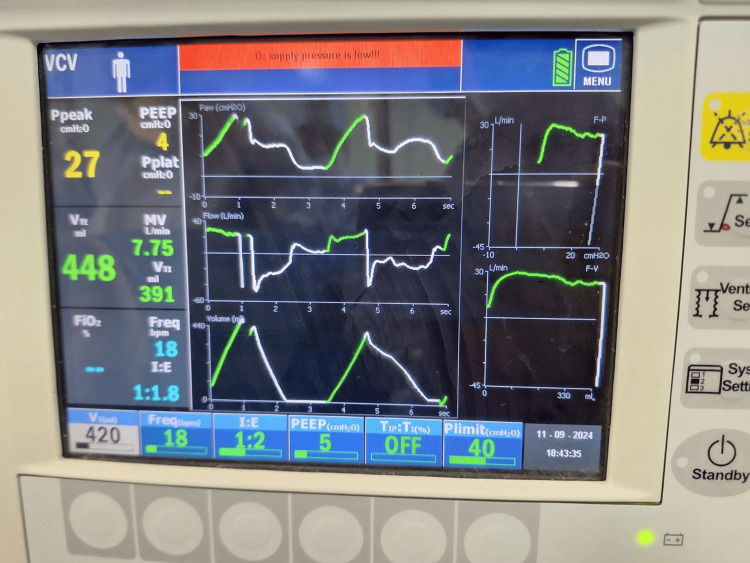
Presence of spontaneous breathing during volume-controlled ventilation

The patient was preoxygenated in a supine position with 100% oxygen for three minutes and premedicated with an injection of 0.2 mg of glycopyrrolate (0.01 mg/kg) + midazolam (1 mg). The patient then was induced with an injection of 120 mg of propofol (2 mg/kg) and paralyzed using an injection of 30 mg atracurium (0.5 mg/kg). The patient was intubated using an endotracheal tube (8.0 size), which was secured after confirming bilateral air entry by auscultation of the lung fields. Under aseptic precautions and ultrasound guidance, a right supraclavicular block was administered via injection of 0.5% bupivacaine (15 mL) + lignocaine (9 mL) + dexamethasone (4 mg). He was positioned in the left lateral position with adequate padding and kept warm with warm IV fluids and a radiant warmer, and his eyes were protected (Figures 1, 2).

**Figure 3:**
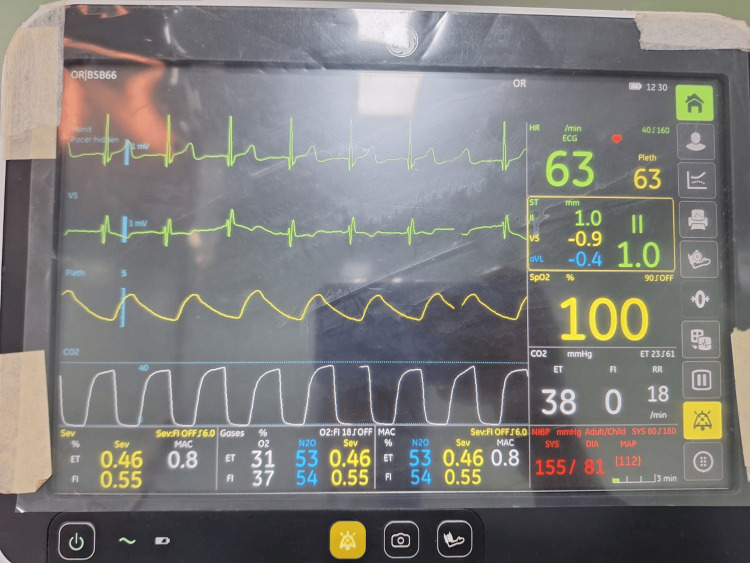
Absence of curare clefts on capnography after achieving adequate depth of anesthesia

Thirty minutes after induction, the patient recovered from the muscle relaxant. Recovery from muscle relaxants was confirmed by the presence of curare clefts in capnography as well as an increase in the heart rate and blood pressure. An injection of 5 mg of atracurium (0.1 mg/kg) was administered, but the patient again exhibited signs of recovery from the muscle relaxant five minutes later confirmed by the presence of curare clefts in capnography as well as an increase in the heart rate and blood pressure. An infusion of dexmedetomidine (0.5 mcg/kg) over 20 minutes along with inhalational agent sevoflurane (one minimum alveolar concentration) was administered, which helped to maintain adequate depth of anesthesia, but then the patient continued to show signs of recovery from muscle relaxant every five minutes. Continuous infusion of dexmedetomidine (0.5 mcg/kg) and boluses of injection atracurium (0.1 mg/kg) were thus administered every 10 minutes with sevoflurane maintained at one minimum alveolar concentration (Figure 3).

The patient then remained hemodynamically stable intraoperatively with no adverse events for the remainder of the three-hour procedure. The total amount of intravenous fluids given was 1500 mL. The total urine output was 500 mL. After achieving adequate respiratory efforts confirmed by a tidal volume of 300 mL (5 mg/kg), as well as muscle power and tone as confirmed by moving of limbs on command, the anesthesia was reversed using an injection of glycopyrrolate (0.01 mg/kg) and neostigmine (0.07 mg/kg) to avoid any residual paralysis or delayed action of the muscle relaxant. Thorough suctioning was done, and the patient was extubated. Post-extubation vocalization was present without stridor. After one hour of monitoring in the recovery room, the patient was transferred to the post-operative ward.

## Discussion

Resistance to neuromuscular blocking agents has been reported in patients with burns and cerebral palsy, as well as in immobilized patients and those on anticonvulsants [2]. These causes were ruled out by taking a detailed history from the patient's son. These conditions were excluded in our patient during his pre-anesthetic checkup. After the patient exhibited frequent emergence from neuromuscular blocking agents during surgery, a drug-drug interaction was suspected. Donepezil was considered the most likely drug responsible for the inadequate neuromuscular blockade.

Donepezil hydrochloride is a centrally acting, rapid, reversible acetylcholinesterase inhibitor. Acetylcholinesterase is an enzyme responsible for terminating neuronal transmission and signaling by breaking down the neurotransmitter acetylcholine into acetic acid and choline. By blocking the enzyme, donepezil increases the availability of acetylcholine at the synapses, thereby increasing cholinergic transmission and improving patients’ cognition and behavior. In this way, donepezil improves symptoms of Alzheimer’s and dementia associated with Parkinson’s disease. For mild to moderate dementia, the initial dosage is 5 mg per day, gradually increasing to 10 mg per day for four to six weeks. Donepezil also shows synergistic effects when combined with other cholinesterase inhibitors (e.g., neostigmine and physostigmine). It prolongs the effects of depolarizing neuromuscular blockers (e.g., suxamethonium) and causes resistance to non-depolarizing neuromuscular blocking agents (e.g., atracurium, rocuronium, and vecuronium) [3].

A 10 mg dose of donepezil has been shown to induce a fourth twitch in Train-of-Four monitoring after a single intubating dose of atracurium, even at the time of intubation [3]. Studies also describe inadequate neuromuscular blockade with rocuronium in patients with Alzheimer’s who take daily donepezil (10 mg). The manufacturer of donepezil and previous studies, therefore, recommend discontinuing the drug two to four weeks before elective surgery [4]. However, a prolonged withdrawal can worsen cognition in these patients, compared to their baseline. Alternatively, maintenance of anesthesia and muscular blockade can be achieved using sevoflurane, dexmedetomidine, and propofol to achieve an adequate plane of anesthesia for surgery. The Naranjo Scale was used to calculate the adverse drug reaction probability, and a score of 7 was obtained. The interpretation is a probable cause of drug interaction between donepezil and neuromuscular blocking agents. 

Currently, there are no clear guidelines on whether donepezil can be continued on the day of surgery or withdrawn before surgery. Abrupt withdrawal can lead to cognitive decline, agitation, delirium, inattentiveness, worsening of sleep habits, and anxiety. Some patients may show delayed worsening, which could be due to the longer half-life of donepezil. In most cases, controlled and gradual tapering to 5 mg per day shows better results [5].

For several reasons, opioid-free anesthesia is becoming a popular peri-operative and post-operative pain management option for many patients, including the elderly. In addition to potential adverse effects caused by drug interactions, many prefer to avoid the side effects of opioids. In elderly patients, these side effects include nausea, constipation, urinary retention, respiratory depression, pruritus, and hyperalgesia [6]. Moreover, due to pharmacokinetic and pharmacodynamic changes in geriatric patients, most drugs are prescribed at lower doses, including opioids, which are usually started at 25%-50% lower doses than usual. Combined with the impaired perception of pain that can occur with dementia, the risk of overdose increases, particularly if the patient cannot communicate well about the nature, severity, and management of their symptoms.

Multimodal analgesia administered during the peri-operative period has been the gold standard for over 20 years [7]. It can replace the use of opioids and their various side effects (e.g., exacerbation of hypotension, respiratory depression, apnea, bradycardia, confusion, urinary retention, constipation, and somnolence), and it can include combinations of drugs like N-methyl-D-aspartate antagonists (e.g., ketamine, lignocaine, and magnesium sulfate), sodium channel blockers (e.g., local anesthetic agents, non-steroidal anti-inflammatory agents, and dexamethasone), and alpha 2 agonists (e.g., dexmedetomidine and clonidine) via regional anesthetic techniques and neuraxial blocks [8,9].

In the case described here, an emergency surgery was performed on an elderly patient with dementia to repair a right-humerus fracture. The patient did not undergo withdrawal of donepezil prior to surgery. During the procedure, drug interaction between donepezil and the neuromuscular blocking agents was evident and managed intraoperatively using opioid-free anesthesia incorporating multimodal analgesia.

## Conclusions

Elderly surgical patients require close monitoring due to age-related physiological changes. Anesthetic management of geriatric patients requires additional knowledge. Common conditions like dementia and other cognitive disabilities are often treated with drugs that can interact with anesthetic agents. Knowledge about treatment protocols, drug interactions, and drug withdrawal protocols is crucial. Opioid use in elderly patients can be detrimental. Hence, opioid-free management of pain via total intravenous or inhalational anesthetics should be considered as a viable, if not preferred, alternative.
